# Autoimmune Myocarditis and Arrhythmogenic Mitral Valve Prolapse: An Unexpected Overlap Syndrome

**DOI:** 10.3390/jcdd8110151

**Published:** 2021-11-06

**Authors:** Andrea Villatore, Simone Sala, Stefano Stella, Davide Vignale, Elena Busnardo, Antonio Esposito, Cristina Basso, Paolo Della Bella, Patrizio Mazzone, Giovanni Peretto

**Affiliations:** 1 Department of Cardiac Electrophysiology and Arrhythmology, IRCCS San Raffaele Scientific Institute, 20132 Milan, Italy; a.villatore@studenti.unisr.it (A.V.); sala.simone@hsr.it (S.S.); dellabella.paolo@hsr.it (P.D.B.); 2 School of Medicine, Vita-Salute San Raffaele University, 20132 Milan, Italy; 3 Myocarditis Disease Unit, IRCCS San Raffaele Scientific Institute, 20132 Milan, Italy; vignale.davide@hsr.it (D.V.); esposito.antonio@unisr.it (A.E.); 4 Department of Echocardiography, IRCCS San Raffaele Scientific Institute, 20132 Milan, Italy; stella.stefano@hsr.it; 5 Experimental Imaging Center, Radiology Unit, IRCCS San Raffaele Scientific Institute, 20132 Milan, Italy; 6 Department of Nuclear Medicine, IRCCS San Raffaele Scientific Institute, 20132 Milan, Italy; busnardo.elena@hsr.it; 7 Department of Cardiac Thoracic Vascular Sciences and Public Health, Cardiovascular Pathology, Padua University, 35128 Padua, Italy; basso.cristina@unipd.it

**Keywords:** myocarditis, ventricular arrhythmias, mitral valve prolapse, arrhythmogenic, sudden cardiac death, endomyocardial biopsy, cardiac magnetic resonance, imaging, ablation

## Abstract

Background: both myocarditis and mitral valve prolapse (MVP) are known uncommon causes of ventricular arrhythmias in young patients. Aim: to report the first clinical case of endomyocardial biopsy (EMB)-proven autoimmune myocarditis and associated arrhythmogenic MVP in a patient with recurrent ventricular fibrillation (VF) episodes. Methods: myocarditis was diagnosed both by cardiac magnetic resonance (CMR) and EMB. Arrhythmogenic MVP was documented by transthoracic echocardiogram, CMR, and electroanatomical mapping of the trigger premature ventricular contractions (PVCs). Results: a 22-year-old woman underwent immunosuppressive therapy after EMB-proven diagnosis of autoimmune myocarditis with VF onset and early implantable cardioverter defibrillator (ICD) placement. Three years later, she experienced two VF recurrences and persistent PVCs, despite no signs of myocarditis recurrence. An echocardiogram revealed bileaflet MVP with high arrhythmic risk features. Finally, electroanatomical mapping and ablation of the trigger PVC were successfully performed. Conclusion: in patients with recurrent VF episodes despite evidence-based medical treatment for myocarditis, MVP should be considered as an alternative arrhythmogenic substrate, and warrants early ICD implant and PVC-targeted therapy.

## 1. Introduction

The prevention of sudden cardiac death in young patients with structural diseases of the myocardium is still a challenge [1,2]. In particular, life-threatening ventricular arrhythmias (VA) have been described in patients with acute myocarditis [3], as well as in those with mitral valve prolapse (MVP) and additional risk features [4]. Consistently with the current expert recommendations [5,6], the diagnostic workup for myocarditis requires endomyocardial biopsy (EMB) to provide definite etiology and allow safe immunosuppressive therapy (IST) [5,7]. Conversely, the diagnosis of MVP primarily relies on transthoracic echocardiogram (TTE). While the long-term arrhythmic risk of myocarditis is still unpredictable [8,9], a number of imaging parameters, including TTE and cardiac magnetic resonance (CMR), have been recently proposed to stratify the MVP arrhythmic risk [10,11].

We report the case of a young woman, initially diagnosed with EMB-proven autoimmune myocarditis and presenting with recurrent VA late after IST withdrawal, who had MVP as an overlapping arrhythmogenic substrate.

## 2. Case Report

A 22-year-old Caucasian woman presented with out-of-hospital cardiac arrest, secondary to ventricular fibrillation (VF). Cardiopulmonary resuscitation was started, with return of spontaneous circulation at 19 min, following five external defibrillation shocks. The patient was subsequently admitted to the Intensive Care Unit. Her past medical history was unremarkable.

**Figure 1:**
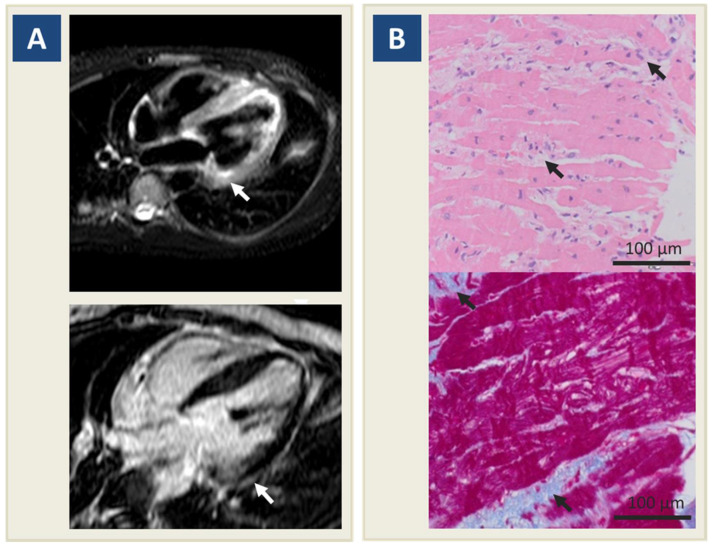
Diagnostic work-up for myocarditis. Legend. (**A**) Cardiac magnetic resonance shows hyperintensity in T2-weighted sequences in basal lateral left ventricular wall (upper panel, arrow) and subepicardial late gadolinium enhancement at the same site (lower panel, arrow). (**B**) Endomyocardial biopsy shows lymphomonocytic inflammatory infiltrates (upper panel, arrows), focal myocyte necrosis, and interstitial edema at hematoxylin eosin assay, associated with spotty replacement fibrosis (lower panel, arrows) at trichrome assay.

On admission, an electrocardiogram (ECG) showed sinus rhythm with normal ST-segment. TTE excluded regional motion abnormalities, and showed no pericardial effusion, nor indirect signs suggesting aortic dissection or pulmonary embolism; however, it showed biventricular systolic dysfunction (left ventricle ejection fraction [LVEF] 45%; tricuspid annular plane systolic excursion (TAPSE) 16 mm) and mitral valve prolapse with mild regurgitation. Baseline electrolytes were within the normal range, but T-troponin was elevated (68 ng L^−1^ with peak at 225 on the same day; n.v. < 14). Urine and blood toxicology tests were normal. To rule out coronary artery disease, a coronary angiography was performed, showing normal epicardial vessels. CMR was performed on day 5, revealing late gadolinium enhancement (LGE) and associated hyperintensity in T2-weighted short-tau inversion recovery (STIR) sequences, in a non-ischemic, subepicardial distribution pattern involving the basal segment of the inferolateral LV wall (Figure 1A). Since the traditional Lake Louise criteria [12] were met, EMB was subsequently planned to confirm the diagnosis of myocarditis and identify etiology, and allowed targeted treatment. By right ventricular septal sampling, a histology report on day 10 documented multiple lymphocytic inflammatory infiltrates (CD3+ > 7/mm^2^), with interstitial edema, focal necrosis, and spots of replacement fibrosis (Figure 1B). Molecular analysis revealed an absence of viral genomes within the myocardial tissue. As is further supported by the identification of circulating anti-intercalated disk and anti-heart autoantibodies in the patient’s serum, a definite diagnosis of autoimmune myocarditis was made. Since frequent polymorphic premature ventricular complexes (PVCs), together with short runs of non-sustained ventricular tachycardia (NSVT) were detected on continuous telemonitoring, a single-chamber transvenous implantable cardioverter defibrillator (ICD) was placed. Before discharge, TAPSE was normal (20 mm), but LV systolic function was still mildly reduced (LVEF 48%). The patient was finally discharged on metoprolol 50 mg bid, flecainide 50 mg bid, and ramipril 2.5 mg. In addition, IST was started, consisting of oral prednisone 1 mg kg^−1^ and azathioprine 2 mg kg^−1^.

**Figure 2:**
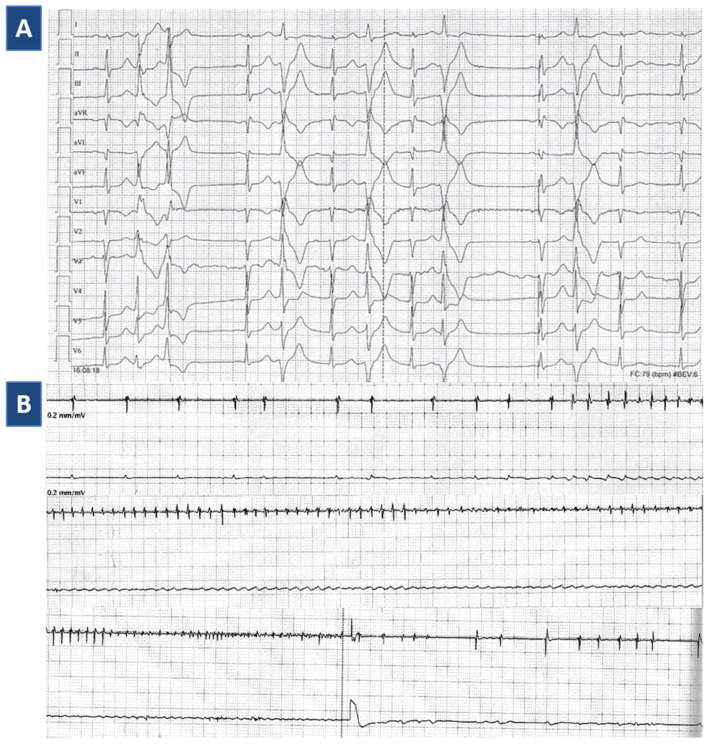
ECG: Ventricular arrhythmias. Legend. (**A**) ECG shows sinus rhythm, with frequent premature ventricular complexes of two morphologies, with bigeminal beats and a couple. The dominant clinical morphology is right-bundle branch block superior axis, suggesting an origin from the inferolateral wall of the left ventricle. (**B**) Ventricular fibrillation episode (49 s, 284 bpm) detected by implantable cardioverter defibrillator and terminated by appropriate shock, three years after immunosuppressive therapy termination.

During follow-up, IST was well tolerated, and no VA were detected by continuous ICD telemonitoring. After 16 months, since the diagnostic workup revealed normal T-troponin and NTproBNP values, LVEF 58% by TTE, and absence of VA induction on treadmill exercise stress test, IST was withdrawn. However, regularly repeated Holter ECGs (4/year) persistently documented high-burden PVCs, in a range of 5000–10,000 daily. PVCs were mainly isolated, with a dominant right-bundle branch block (RBBB) superior axis morphology (Figure 2A). In addition, early ectopies, as well as couplets, triplets, and bigeminal phases, were frequently documented, preventing the safe withdrawal of betablocker and anti-arrhythmic treatment.

**Figure 3:**
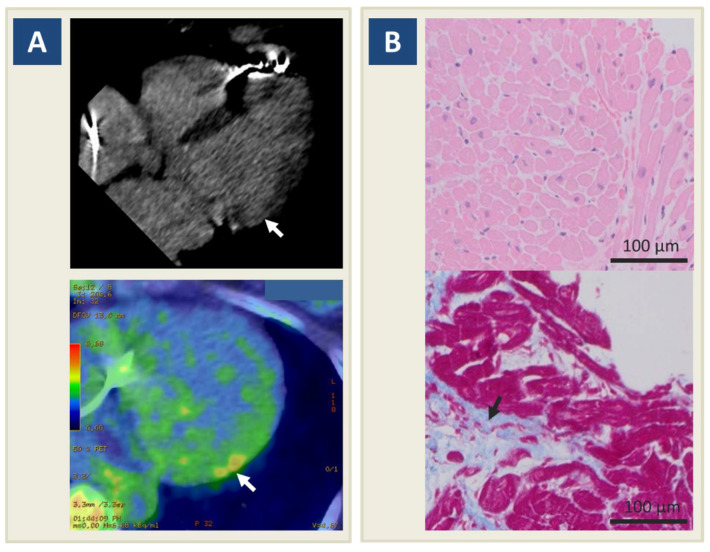
Follow-up restaging of myocarditis. Legend. (**A**) Cardiac computed tomography shows subepicardial late iodine enhancement in the lateral left ventricular wall (upper panel, arrow), corresponding to baseline findings on cardiac magnetic resonance; at the same time, 18-F fluorodeoxyglucose positron emission tomography scan shows no remarkable myocardial inflammatory activity at the same site (lower panel, arrow). (**B**) Follow-up endomyocardial biopsy shows rare lymphocytic inflammatory cells (CD3+ < 7 mm^2^), lack of edema and no cardiac myocyte necrosis (upper panel); fibrosis is the dominant component (lower panel, arrow).

**Figure 4:**
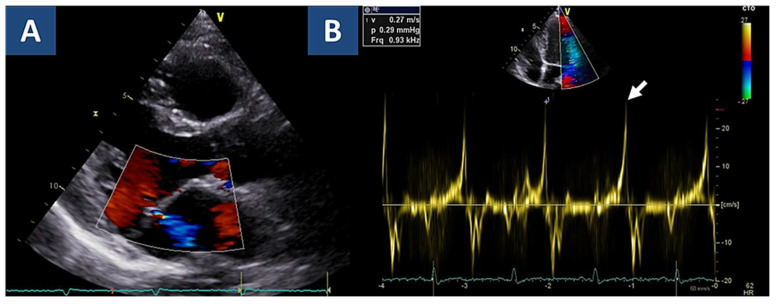
Echocardiographic findings. Legend. (**A**) Parasternal long axis view shows mild mitral valve regurgitation. Associated curling of the basal inferolateral wall is shown as Supplementary Video. (**B**) As a hallmark of increased arrhythmic risk, Pickelhaube sign (spiked systolic lateral mitral annular velocity >16 cm/s) is shown (arrow).

After three years of clinical stability, the patient experienced two episodes of VF appropriately identified and interrupted by ICD shock (Figure 2B). On suspicion of arrhythmic myocarditis relapse, she was immediately admitted to hospital for clinical reassessment. Since follow-up CMR was limited by ICD-related artifacts, a fluorodeoxyglucose positron emission tomography/computed tomography (18F-FDG PET/CT) scan was ordered: in particular, CT confirmed subepicardial LV basal inferolateral late iodine enhancement consistently with the prior myocarditis, whereas 18F-FDG PET showed non-specific LV capitation, suggesting a low probability of myocarditis recurrence (Figure 3A). Consistently, T-troponin was normal. To definitely rule out a recurrence of myocarditis, a new EMB was performed, documenting spots of replacement fibrosis with lack of inflammatory infiltrates (Figure 3B). In the absence of consistent treatment targets, the indication to a new IST cycle was excluded following multidisciplinary reassessment [13]. Instead, a repeated echocardiogram provided new insights in characterizing the known MVP: in particular, thickened valvular leaflets consistent with Barlow disease were noted, together with basal wall systolic curling (Supplementary Video), and a Pickelhaube sign [14], i.e., elevated systolic lateral mitral annular velocity (27 cm/s, n.v. < 16) with a typical spiked S wave (Figure 4). The patient was started on amiodarone, which was withdrawn after one week because of significant QT prolongation (QTc 510 ms).

**Figure 5:**
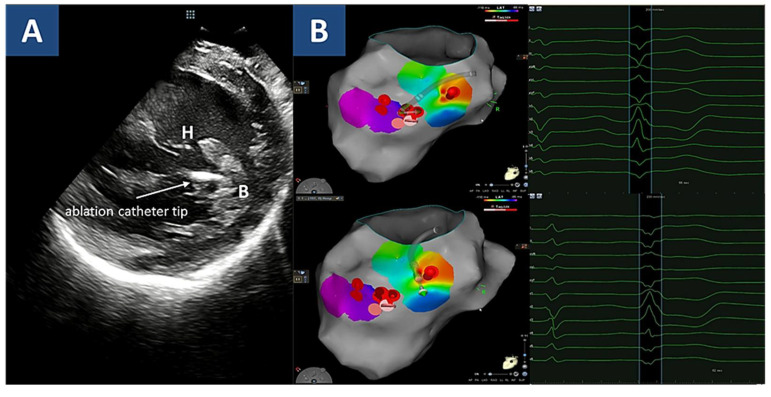
Mapping and ablation. Legend. (**A**) Intracardiac echography shows the target sites for radiofrequency ablation. In particular, the most common premature ventricular complex morphology originated from the posterior papillary muscle basis (B), whereas the second one, emerging after the targeting of the basal site, arose from the head (H) of the papillary muscle. Ablation catheter tip is shown (arrow). (**B**) activation map for both PVC morphologies is shown. Radiofrequency delivery sites appear as red dots at basis (upper panel) and head (lower panel) of the posterior papillary muscle.

Given the persistence of frequent PVCs, the patient was finally scheduled for a catheter ablation procedure: under intracardiac echography (ICE) guidance, the LV endocardial geometry was acquired by electroanatomical mapping (CARTO^®^). Two PVC morphologies were detected and mapped, both showing a RBBB superior axis (Figure 5): the clinical and most frequent one originated from the basis of the posterior papillary muscle (PPM), as confirmed by 12/12 match on pacemapping; the second one, which emerged later during the procedure, arose from the head of the PPM and showed shorter coupling intervals. RF pulses were successfully delivered at both sites, and absent VA inducibility was documented by an end-procedure programmed ventricular stimulation test. The patient was finally discharged without anti-arrhythmic treatment. At a 6-month follow-up from ablation, she was asymptomatic, free from VA, and with a significant reduction in PVC burden (800/24 h, with no evidence of any of the targeted morphologies).

## 3. Discussion

We presented, to our knowledge, the first case of autoimmune myocarditis and MVP coexisting as arrhythmogenic substrates in a young woman with recurrent VA. Although the diagnosis of MVP was known since her first clinical presentation, the initial diagnostic workup was strongly suggestive of myocarditis. In particular, myocardial inflammatory disease was initially pointed out by the classic Lake Louise at CMR [12], and then confirmed by EMB findings, allowing definite diagnosis of virus-negative lymphocytic myocarditis [5,6]: in keeping with an autoimmune disease, circulating autoantibodies were also identified in the patient serum [5,15]. Remarkably, the EMB-proven diagnosis was the cornerstone to allow the safe use of IST, as a proven effective treatment for both non-arrhythmic and arrhythmic myocarditis [16,17]. The subsequent documentation of VF episodes without clear signs of myocarditis recurrence was completely unexpected, and constituted a remarkable turning point in the patient’s workup. In fact, although the patient has been known for MVP since first evaluation, a comprehensive characterization for arrhythmic risk was performed only at that point. Aside from leaflet morphological features consistent with Barlow disease, additional risk features were identified, including the Pickelhaube sign [14]. These findings were also in keeping with the origin of early PVC from the PPM, as documented by electroanatomical mapping.

Overall, the electrocardiographic analysis of VA was consistent with both the diagnoses. In fact, it has been demonstrated that polymorphic and irregular VA are usually associated with active-phase myocarditis, in contrast with healed myocarditis [18,19]. In turn, the dominant PVC morphology was RBBB with superior axis, as in most cases with myocarditis [18,19]. On the other hand, it is accepted knowledge that VF episodes in MVP patients are triggered by early PVCs often arising from the PPM, and therefore displaying a similar RBBB superior axis morphology [4,20]. Since electroanatomical mapping currently allows a bidimensional assessment, ICE was needed to differentiate the PPM rather than basal inferolateral free wall location as the origin of the clinical PVC as a known trigger for VF and suitable target for ablation [21,22,23,24].

Concerning risk stratification, the patient was initially implanted with an ICD. In fact, although the current guidelines suggest refraining from a secondary prevention cardiac device implant until myocarditis healing, the patient had persistent, symptomatic episodes of NSVT, premature PVCs, and anti-intercalated disks autoantibodies as an additional hallmark of arrhythmic risk [17,25]. Retrospectively, the strategy was beneficial, and fully fitting with the recommendation for secondary prevention ICD implant in patients with arrhythmogenic MVP [1,2]. Furthermore, several predictors of malignant VA were found in association with MVP, including female gender, bileaflet MVP, LGE on CMR, and complex PVCs [26]. A Pickelhaube sign was an additional hallmark of increased risk, and furtherly supported the indication to ICD implant.

Assuming independent events, the coexistence of two relatively uncommon arrhythmogenic diseases in a single patient is undoubtedly exceptional. However, we identified some remarkable clues to speculate about a possible link between the conditions: the patient had no history of viral infections, exposure to toxic agents, or systemic autoimmune diseases [5,27]. In the absence of an alternative identifiable trigger, the initiator of cardiac-specific autoimmune response directed against multiple molecular players, namely sarcomeric proteins and intercalated disks released by injured myocytes [5,15], may have been intrinsic to the MVP pathophysiology. In fact, there is a strict mechanical connection between the PPM stretching and the basal inferolateral wall motion abnormalities and local injury [4]. Consistently, association between MVP and myocarditis was previously reported in another clinical case with documented VA [28], and lymphocytic inflammatory infiltrates were described in postmortem studies on MVP patients experiencing arrhythmic sudden death [4]. Although definite criteria for autoimmune myocarditis [5] have not been reported so far, it may be hypothesized that the subacute or chronic myocardial inflammation is primarily triggered by the PPM stretching and associated LV inferobasal wall motion abnormalities. It should be noted that although MVP is not conventionally defined as a cardiomyopathy, myocardial inflammation and associated tissue abnormalities do widely overlap with those reported in many other non-ischemic diseases [29,30,31]. The described clinical case showed consistency with this hypothesis, both in the spatial domain, given the basal inferolateral LV wall involvement shared between myocarditis and MVP, with an overall consistent dominant PVC morphology [20], and in the temporal domain, since replacement fibrosis was documented even at the initial EMB, hinting at a non-acute process [18,25,27] despite the abrupt clinical onset. Confirmatory evidence is undoubtedly needed in this area, and would provide novel mechanistic insights into arrhythmogenic MVP pathophysiology. Additionally, since myocardial inflammation was successfully targeted by IST and no arrhythmic events were detected until three years later, novel therapeutic options could be applied to target arrhythmogenic MVP in the future.

The limitations of this case report include lack of direct documentation of an arrhythmogenic role for the targeted PVC morphologies, the impossibility of obtaining an informative follow-up CMR for further tissue characterization, the lack of substrate-based EMB sampling, and the short-term follow-up.

## 4. Conclusions

In conclusion, we showed that myocarditis and arrhythmogenic MVP can manifest in a single patient. Although a direct connection between the diseases cannot be hereby established, we reported late VF episodes despite evidence-based treatment for myocarditis and no convincing signs of persistent activity. This experience suggests that if myocarditis and MVP coexist, secondary prevention ICD should be considered.

## Data Availability

Additional data will be made available, upon reasonable request, by contacting the corresponding author via email.

## Supplementary Materials

The following are available online at https://www.mdpi.com/article/10.3390/jcdd8110151/s1, Supplementary Video.
